# Genetic Background, Adipocytokines, and Metabolic Disorders in Postmenopausal Overweight and Obese Women

**DOI:** 10.1007/s10528-016-9743-z

**Published:** 2016-05-31

**Authors:** Bogna Grygiel-Górniak, Elżbieta Kaczmarek, Maria Mosor, Juliusz Przysławski, Anna Bogacz

**Affiliations:** 1 Department of Bromatology and Human Nutrition, Poznan University of Medical Sciences, Poznan, Poland; 2 Department of Rheumatology and Internal Diseases, Poznan University of Medical Sciences, Poznan, Poland; 3 Department of Bioinformatics and Computational Biology, Poznan University of Medical Sciences, Poznan, Poland; 4 Department of Molecular Pathology, Institute of Human Genetics, Polish Academy of Sciences, Poznan, Poland; 5 Laboratory of Experimental Pharmacogenetics, Department of Clinical Pharmacy and Biopharmacy, Poznan University of Medical Sciences, Poznan, Poland

**Keywords:** Postmenopausal obesity, *PPAR gamma*-*2* and *beta3*-*AR* polymorphism, Adipocytokines, Metabolic disorders, Body fat distribution

## Abstract

The relationship between the genetic background, adipocytokines, and metabolic state in postmenopausal women has not yet been fully described. The aim of this study was to determine the relationship between *PPAR gamma*-*2* (Pro12Ala, C1431T) and *ADRB3* (Trp64Arg) polymorphisms and serum adipocytokines (adiponectin, visfatin, and resistin) and metabolic disorders in 176 postmenopausal women with increased body mass (BMI ≥ 25 kg m^−2^). The distributions of selected alleles and genotype frequencies were determined with the PCR–RFLP method. The bioimpedance method was used to determine nutritional status, and enzyme-linked immunosorbent assays were applied to determine serum concentrations of adipocytokines. Viscerally obese postmenopausal women had higher body mass, body fat content, serum glucose, insulin, total cholesterol, LDL, triglycerides, uric acid, and HOMA-IR and a higher prevalence of the Ala12 allele. In models based on cytokine concentration, higher body mass and glucose concentration (visfatin model, *p* = 0.008) and higher insulin and triglyceride levels (resistin model, *p* = 0.002) were observed in visceral fat deposition and this was potentiated by the presence of the T1431 allele. In resistin models, co-existence of Ala12/X polymorphisms with the T1431 allele was associated with higher resistin and triglyceride concentrations (*p* = 0.045). In postmenopausal women, metabolic parameters are mainly determined by the distribution of body fat, but Ala12/X polymorphism may increase the metabolic disorders and this effect can be enhanced by the T1431 allele.

## Introduction

The Pro12Ala variant of *PPAR gamma*-*2* gene is associated with obesity (Yao et al. 2015), diet (Luan et al. 2001), body mass regain after dieting (Nicklas et al. 2001). The gene, which strongly interacts with Pro12Ala in determining body mass, is the beta3-adrenoceptor gene—*ARDB3* (Trp64Arg polymorphism) (Hsueh et al. 2001). *PPAR gamma*-*2,* as a transcription factor, modulates the expression of several genes encoding adiponectin, visfatin, and resistin, which are involved in fatty acid metabolism, glucose homeostasis, and insulin sensitivity (Steppan et al. 2001; Maeda et al. 2001; Patel et al. 2003; Mayi et al. 2010). These adipocytokines have a crucial influence on metabolic disorders. Adiponectin increases insulin sensitivity by enhancing fatty acid oxidation and glucose uptake in skeletal muscle and adipose tissue, and decreases hepatic glucose release (Pittas et al. 2004; Iwaki et al. 2003). Hipoadiponectinemia is observed in obese (Arita et al. 1999) and diabetic patients (Weyer et al. 2001). Visfatin is mainly produced and secreted by visceral adipocytes and causes insulin-mimetic effects by binding to the insulin receptor (Revollo et al. 2007). Resistin is considered to link obesity to insulin resistance, and is expressed in adipocytes and macrophages (Steppan et al. 2001). Moreover, many changes in metabolic parameters and adipocytokine concentrations depend on visceral fat distribution (Arita et al. 1999; Tokunaga et al. 2008; Cullen 2000), which is related with increased morbidity of cardiovascular diseases (Cullen 2000; Rosenquist et al. 2015). Visceral adipose tissue is more metabolically active than subcutaneous adipose tissue and is of greater importance for the development of obesity-related complications (Rosenquist et al. 2015).

Although polymorphisms of *PPAR gamma*-*2* and *ARDB3* genes are considered to be closely related in numerous populations with susceptibility to obesity and diabetes mellitus type 2, no data have been reported for specific relationships with body mass, glucose, lipid profile, or adipocytokine concentrations in postmenopausal women. In view of the contentious association of *PPAR gamma*-*2* and *ARDB3* genes with glucose and lipid disorders, the present study was designed to evaluate the influence of the analyzed polymorphisms (Pro12Ala rs1801282, C1431T rs3856806, and Trp64Arg rs4994) on cytokine levels and metabolic disorders in postmenopausal women with increased body mass.

## Materials and Methods

A total of 877 postmenopausal women were selected from the Metabolic Outpatient Clinic and invited to undergo anthropometrical measurements. From this group, women without essential diseases such as non-treated thyroidal disorders, acute liver and renal diseases, neoplasm diagnosed during last 5 years, cardiovascular diseases (with the exception of hypertension), acute infections, smoking, or taking vitamins or mineral supplements participated in the study. Subjects receiving concomitant medication, including lipid lowering and hypoglycemic medications, were excluded from this analysis. Finally, an examination and interview with a doctor enabled 176 women to be selected for the study. All subjects enrolled in this study provided their written informed consent. This study was approved by the local Bioethics Committee of Poznan University of Medical Sciences (no. 792/09) and was performed according to the Helsinki Declaration.

### Anthropometric Measurements

Anthropometric measures included height, weight, and waist and hip circumference. Weight was measured to the nearest 0.1 kg using digital scales, while the subjects were minimally clothed, without shoes. Height was measured using a vertical ruler to the nearest 0.5 cm. Waist circumference (WC) was measured to the nearest 0.1 cm, midway between the lower border of the ribs and the iliac crest at the widest portion, over light clothing, using a soft measuring tape, without any pressure to the body. Body mass index was calculated as weight/height squared (kg m^−2^) and waist-to-hip ratio (WHR) as the proportion of waist-to-hip circumferences [a high WHR > 0.85 in women indicated visceral (abdominal) fat accumulation] (WHO 2000). All anthropometric components were measured twice by study staff using a standardized protocol and averaged. Derived variables enabled the calculation of waist-to-hip ratio and BMI. The body fat (FM) content and lean body mass (LBM) was assessed by bioimpedance method using BODYSTAT 1500—a single-frequency (50 kHz) device (Bodystat Ltd. Isle of Man, UK).

### Biochemical Analysis

Blood samples were collected between 7:00 a.m. and 8:00 a.m. after an overnight fast. Venous blood samples were collected in EDTA-containing tubes, which were immediately centrifuged. Plasma glucose and lipid profile [total cholesterol (TC), high-density lipoprotein (HDL), and triglycerides (TG)] and uric acid were measured using enzymatic colorimetric assays (Cobas Integra 400 Plus; Roche Diagnostics, Indianapolis, IN). Low-density lipoprotein (LDL) was calculated from serum TC, TG, and HDL according to the Friedewald equation (Friedewald et al. 1972). FSH serum levels were measured via specific chemiluminescence assays from Roche Diagnostic. Plasma insulin levels were determined by means of an enzymatic immunoassay [Cobas Integra 400 Plus; Roche Diagnostics]. Insulin resistance was estimated by Homeostasis Model Assessment (HOMA), according to the formula: HOMA-IR = fasting plasma glucose (mmol^−l^) × fasting insulin (mU L^−1^)/22,5. Enzyme-linked immunosorbent assays (ELISA) were used to determine serum concentrations of adiponectin (Human Total Adiponectin/Acrp30 Quantikine ELISA Kit, BIOKOM), visfatin (Visfatin human Elisa, DRG MedTek), and resistin levels (Human Resistin Quantikine ELISA Kit, BIOKOM) in fasting venous blood samples collected from the patients. All the plasma levels of adipocytokines were measured in strict accordance with the manufacturer’s instructions.

### Genotyping

Genomic DNA isolation from venous blood samples was performed according to the manufacturer’s protocol (Gentra Puregene Blood Kit, Qiagen, Germany). Analysis of the Pro12Ala (rs1801282) polymorphism of PPAR gamma-2 was determined using the TaqMan genotyping assay (C_1129864_10) (Applied Biosytsem, Foster City, US). Reaction mixture for the determination of Pro12Ala polymorphism contained the forward target-specific polymerase chain reaction primer, the reverse primer, and the TaqMan MGB probes labeled with two special dyes (FAM and VIC) and 15 ng of each sample. The thermal cycling conditions were the following: 10 min at 95 °C, 40 cycles of 92 °C for 15 s and 60 °C for 1 min. Genotyping was performed using ABI7900HT (Applied Biosystems, Foster City, CA, USA).

Determination of the C1431T (rs3856806) polymorphism of PPAR gamma-2 was performed using the polymerase chain reaction and restriction fragment length polymorphism (PCR–RFLP). The 170 bp PCR product was digested with *Eco72I* enzyme according to the manufacturer’s instruction (Fermentas, Vilnius, Lithuania). The PCR product of wild-type DNA generated fragments of 127 and 43 bp, but fragment DNA did not cut for mutant DNA. Products of the electrophoresis were evaluated by visualization in the UV light using 2.5 % agarose gels.

The analysis of the Trp64Arg (rs4994) polymorphism of *ADRB3* gene was performed using the PCR method as described by Sivenius et al. (2000). Genotypes of the Trp64Arg polymorphism were determined by a TaqMan genotyping assay (C 2215549_20) (Life Technologies, Carlsbad, Calif). The reaction was performed with HOT FIREPol Probe qPCR Mix Plus (no ROX) according to the manufacturer’s instructions provided by Solis Biodyne (Tartu, Estonia). Genotyping analysis was done using a CFX96 TouchT Real-Time PCR Detection System (Bio-Rad, Hercules, California, U.S). The PCR thermal cycling was as follows: initial denaturation at 95 °C for 15 min; 40 cycles of 95 °C for 15 s and 60 °C for 60 s. As a quality control measure, negative controls and approximately 5 % of samples were genotyped in duplicate to check genotyping accuracy.

### Genetic Analysis

The genotype data were used to construct the haplotypes between the two polymorphisms using Haploview 4.2 software to evaluate the linkage disequilibrium (LD). LD between the SNPs used in haplotype analysis was measured using a pairwise D′ statistic. The structure of the LD block was examined with the method proposed by Gabriel et al. using the 80 % confidence bounds of D′ to define sites of historical recombination between SNPs (Gabriel et al. 2002). The haplotype frequencies were calculated based on the maximum likelihood method with Haploview 4.2 software.

### Statistical Analysis

The distribution of selected genotypes and allele frequencies was analyzed using *χ*^2^ test or Fisher’s exact test (for small frequencies) with odds ratios (OR). The expected frequencies of genotypes were determined by Hardy–Weinberg equilibrium. The Shapiro–Wilk test was used in order to determine whether the continuous variables were normally distributed. Continuous data were shown in the tables as mean ± SD. The unpaired *t*-test was used to verify the hypothesis that differences between each analyzed anthropometric, biochemical, metabolic factors, and cytokine levels in the analyzed groups were significant. To analyze whether the level of the studied cytokines is associated with categorical factors and continuous predictors, generalized linear models were studied (Hill and Lewicki 2007; Green and Silverman 1994).

Effects of continuous (anthropometric measurements biochemical and metabolic data) and categorical predictor variables (polymorphisms, visceral, and gynoidal body fat distribution) on a continuous-dependent variable (level of visfatin and resistin) were analyzed using log linear models with a likelihood test based on a logarithmic link function. We decided to perform the generalized linear model in our studies because the distribution of the dependent variable does not have to be normal and continuous, and the values of the dependent variable are predicted from a linear combination of predictor variables, which are connected to the dependent variable via a link function while in the general linear model values of the dependent variable should have normal distribution and the link function is a simple identity function. It means that a linear combination of values for the predictor variable is not transformed. For categorical predictor variables, we can fit ANOVA-like designs. Designs can be incomplete. The indicator variable represents effects for categorical predictor variables.

The power of the test used to detect hypothetical differences between the analyzed groups was also determined. The statistical analysis was performed with Statistica v. 10.0 (StatSoft Inc.); *p*-value of <0.05 indicates significant results.

## Results

The differences between genotype distributions and allele frequencies for the Pro12Ala, C1431T, and Trp64Arg polymorphisms between groups with visceral (V) and gynoid (G) fat distributions were statistically insignificant with the exception of the distribution of CC genotypes as well as C and G alleles for Pro12Ala polymorphism (Table 1). The observed genotype frequencies of polymorphisms were all in agreement with the Hardy–Weinberg equilibrium.

**Table 1:** Genotype and allele frequencies of the Pro12Ala and C1431/X *PPAR gamma*-*2* and Trp64Arg of *beta*-adrenergic receptor gene polymorphisms according to gynoid (WHR < 0.85) and visceral fat distribution (WHR > 0.85)

Body fat distribution	Gynoid (*n* = 95)		Visceral (*n* = 81)		OR	95 % CI	*p*-value
	Observed *n* (%)	Expected (%)	Observed *n* (%)	Expected (%)			
Genotype (Pro12Ala)							
CC	69 (72.63)	72.69	48 (59.26)	58.58	1.82	0.92−3.61	0.043
CG	24 (25.26)	25.14	28 (34.57)	35.91	0.64	0.32−1.29	0.118
GG	2 (2.11)	2.17	5 (6.17)	5.51	0.32	0.03−2.08	0.161
Allele							
C	162 (85.26)	–	124 (76.54)	–	1.77	0.99−3.17	0.025
G	28 (14.74)	–	38 (23.46)	–	0.56	0.32−1.01	0.026
Genotype (C1431T)							
CC	64 (67.37)	69.16	60 (74.07)	70.48	0.72	0.35−1.46	0.210
CT	30 (31.58)	28.01	16 (19.76)	26.94	1.87	0.89−4.05	0.053
TT	1 (1.05)	2.83	5 (6.17)	2.58	0.16	0.01−1.50	0.072
Allele							
C	158 (83.16)	–	136 (83.95)	–	0.94	0.51−1.73	0.470
T	32 (16.84)	–	26 (16.05)	–	1.06	0.58−1.95	0.480
Genotype (Trp64Arg)							
TT	79 (83.16)	81.96	66 (81.48)	82.33	1.12	0.47−2.62	0.460
TC	14 (14.74)	17.15	15 (18.52)	16.81	0.76	0.32−1.83	0.320
CC	2 (2.10)	0.89	–	0.86	–	–	–
Allele							
T	172 (90.53)	–	147 (90.74)	–	0.97	0.44−2.13	0.540
C	18 (9.47)	–	15 (9.26)	–	1.03	0.47−2.26	0.550

Data are *n* (%) for genotypes and *n* (frequency) for alleles

*Pro12Ala* and *C1431T* polymorphisms of *PPAR gamma*-*2* gene, *Trp64Arg* polymorphism of *ADRB3* gene, *OR* odds ratio, *CI* confidence interval

The age, height, and lean body mass of the analyzed women were similar (Table 2); however, body mass and body fat content were higher in the group with visceral fat distribution and this indicated obesity (BMI ≥ 30 kg m^−2^), while gynoid subjects were overweight. The concentrations of FSH and HDL levels were higher in women with gynoid fat deposition (BMI = 25–30 kg m^−2^). Glucose, insulin, HOMA-IR, TC, LDL, TG, and uric acid were elevated in viscerally obese women. The levels of adipocytokines did not differ between gynoid and visceral groups. The analysis of the association between polymorphisms and serum uric acid concentration (data not shown in tables) revealed significant increase of uric acid level (*p* = 0.0495) in the Ala12/X group (mean 5.07 ± 1.11 mg dL^−1^) when compared to Pro12Pro group (mean 4.73 ± 1.08 mg dL^−1^). After adding the standard deviation to the mean value of uric acid level in women with Ala12/X polymorphism, it exceeded the reference value for uric acid, while in subjects with Pro12Pro polymorphism uric acid was within the normal range. Serum uric acid level did not differ between the subgroups with Trp64Arg and C1431T polymorphisms.

**Table 2:** Anthropometric and metabolic characteristics of analyzed women according to body fat distribution (gynoid vs. visceral)

Analyzed parameters	Body fat distribution		
	Gynoid (*n* = 95)	Visceral (*n* = 81)	*p*-value
Age (years)	59.01 ± 6.12	59.31 ± 5.50	0.736
Height (cm)	161.70 ± 6.32	160.40 ± 5.17	0.139
Body mass (kg)	72.37 ± 16.82	82.93 ± 16.04	<0.001
BMI (kg m^−2^)	27.51 ± 6.45	32.31 ± 5.94	<0.001
Waist circumference (cm)	83.37 ± 12.33	100.69 ± 11.05	<0.001
Body fat (% of body mass)	41.28 ± 7.04	46.36 ± 5.78	<0.001
Body fat (kg)	30.59 ± 12.25	39.11 ± 11.39	<0.001
LBM % (% of body mass)	58.65 ± 7.17	53.87 ± 5.69	<0.001
LBM (kg)	42.00 ± 9.77	48.39 ± 40.37	0.139
BMR (kcal day^−1^)	1344.93 ± 146.79	1397.83 ± 131.72	0.014
FSH (mIU mL^−1^)	72.59 ± 27.27	62.77 ± 23.88	0.013
Glucose (mg dL^−1^)	94.29 ± 10.35	100.09 ± 16.02	0.004
Insulin (mU mL^−1^)	7.71 ± 3.92	11.76 ± 7.01	<0.001
HOMA-IR (mmol × mU L^−2^)	1.84 ± 1.13	2.97 ± 1.93	<0.001
Total cholesterol (mg dL^−1^)	227.01 ± 43.97	242.20 ± 42.79	0.022
HDL (mg dL^−1^)	67.80 ± 16.22	60.21 ± 11.53	0.001
TG (mg dL^−1^)	100.95 ± 41.10	145.52 ± 57.98	<0.001
LDL (mg dL^−1^)	139.22 ± 38.69	152.83 ± 39.15	0.022
Uric acid (mg dL^−1^)	4.55 ± 0.95	5.18 ± 1.16	<0.001
Resistin (ng mL^−1^)	2.90 ± 3.06	3.19 ± 3.86	0.600
Adiponectin (µg mL^−1^)	15.93 ± 9.46	15.88 ± 9.04	0.973
Visfatin (ng mL^−1^)	4.50 ± 1.77	4.46 ± 1.83	0.911

*BMI* body mass index, *LBM* lean body mass (expressed in kg and percent of body fat), *BMR* basal metabolic rate, *FSH* follicle-stimulating hormone

Because of the very low number of Ala12Ala genotypes at Pro12Ala polymorphism, data from Pro12Ala and Ala12Ala (i.e., Ala12/X) individuals were pooled and analyzed together (Table 3). Similarly, T1431T and C1431T as well as Trp64Arg and Arg64Arg were pooled and analyzed as T1431/X and Arg64/X, respectively. Women with Arg64/X polymorphism were characterized by higher levels of resistin, while higher concentrations of adiponectin and lower ones of visfatin were observed in the group with BMI < 30 kg m^−2^.

**Table 3:** Cytokine levels in analyzed polymorphisms and the amount/distribution of body fat

Analyzed polymorphisms	Pro12Pro (*n* = 117)	Ala12/X (*n* = 59)	*p*
Adiponectin (µg mL^−1^)	16.11 ± 9.04	15.49 ± 9.70	0.672
Visfatin (ng mL^−1^)	4.54 ± 1.94	4.38 ± 1.96	0.608
Resistin (ng mL^−1^)	2.74 ± 2.94	3.63 ± 4.29	0.133

Analyzed polymorphisms	Trp64Trp (*n* = 142*)*	Arg64/X (*n* = 32)	*p*
Adiponectin (µg mL^−1^)	15.73 ± 9.08	16.55 ± 10.0	0.651
Visfatin (ng mL^−1^)	4.60 ± 2.05	3.96 ± 1.29	0.094
Resistin (ng mL^−1^)	2.76 ± 2.95	4.46 ± 5.14	0.022

Analyzed polymorphisms	C1431C (*n* = 124*)*	T1431/X (*n* = 52)	*p*
Adiponectin (µg mL^−1^)	15.88 ± 8.82	15.94 ± 10.26	0.967
Visfatin (ng mL^−1^)	4.49 ± 1.94	4.47 ± 1.96	0.960
Resistin (ng mL^−1^)	2.79 ± 3.05	3.64 ± 4.25	0.167

BMI value	BMI < 30 kg m^−2^ (*n* = 74)	BMI ≥ 30 kg m^−2^ (*n* = 102)	*p*
Adiponectin (µg mL^−1^)	17.68 ± 9.78	14.61 ± 8.64	0.029
Visfatin (ng mL^−1^)	4.08 ± 1.87	4.77 ± 1.94	0.019
Resistin (ng mL^−1^)	2.72 ± 2.12	3.23 ± 4.05	0.372

Distribution of body fat	Gynoid (*n* = 95*)*	Visceral (*n* = 81)	***p***
Adiponectin (µg mL^−1^))	15.93 ± 9.46	15.87 ± 9.04	0.973
Visfatin (ng mL^−1^)	4.50 ± 2.04	4.46 ± 1.83	0.911
Resistin (ng mL^−1^)	2.90 ± 3.06	3.19 ± 3.86	0.600

In Table 4, we analyzed the level of visfatin and resistin concentration in the context of the polymorphisms present and the body fat distribution (Table 4). In a visfatin model (*p* = 0.008), a higher body mass and glucose concentration was observed in visceral fat deposition. The co-existence of visceral fat distribution with the T1431 allele was associated with the highest body mass and glucose level (*p* < 0.05). Analysis of the resistin concentration showed that the differences in metabolic parameters (glucose, insulin, and TG) in four groups of patients with C1431T variants were mainly determined by the visceral deposition of body fat. The highest levels of insulin, glucose, and TG were present in viscerally obese women with the T1431 allele (statistical significance of the model *p* = 0.002).

**Table 4:** Visfatin, resistin, and statistically significant metabolic characteristics in the analyzed group of C1431T and T143 polymorphisms and body fat distribution

Analyzed polymorphism	Type of obesity	*n*	Adipocytokines		Metabolic characteristics			
			Visfatin	Resistin	Body mass (kg)	Glucose (mg dL^−1^)	Insulin (mU dL^−1^)	TG (mg dL^−1^)
			*X* ± SD	*X* ± SD	*X* ± SD	*X* ± SD	*X* ± SD	*X* ± SD
C1431C	Gynoidal	G1 (*n* = 57)	4.3 ± 1.86	2.55 ± 2.53	75.13 ± 15.79	95.87 ± 11.64	8.19 ± 4.14	107.9 ± 43.5
	Visceral	G2 (*n* = 58)	4.62 ± 1.99	3.05 ± 3.55	82.22 ± 15.21	98.45 ± 16.43	11.13 ± 5.6	139.1 ± 54.9
T1431	Gynoidal	G3 (*n* = 29)	4.74 ± 2.09	3.67 ± 3.95	68.69 ± 17.98	92.04 ± 8.1	7.53 ± 3.75	91.9 ± 35
	Visceral	G4 (*n* = 19)	3.97 ± 1.33	3.6 ± 4.73	83.26 ± 17.87	105.57 ± 14.91	13.53 ± 9.83	171.1 ± 75.1
Comparisons between groups: *p < 0.05, ** *p* < 0.01, *** *p* < 0.001			ns	ns	G2 vs. G3* and G3 vs. G4*	G1 vs. G4* and G3 vs. G4**	G3 vs. G4*	G1 vs. G2* and G1 vs. G4*** and G2 vs. G3** and G3 vs. G4***
Power of the test			<50 %	<50 %	83 %	80 %	50 %	80 %
Significant association (sv) between visfatin, metabolic parameters, analyzed polymorphism and type of obesity (*p* = 0.008)			sv	–	sv	sv	–	–
Significant association (sr) between resistin, metabolic parameters, analyzed polymorphism and type of obesity (*p* = 0.002)			–	sr	–	sr	sr	sr

* *p* < 0.05; ** p < 0.01; *** p < 0.001

More advanced models included analysis of resistin levels and two polymorphisms: Pro12Ala with C1431T (*p* = 0.045) and Trp64Arg with C1431T (*p* = 0.027) (Table 5). In both models, insulin and triglyceride concentrations were higher in the visceral distribution of fat of all analyzed polymorphisms. The highest level of TG was observed in visceral women with Ala12/X and T1431/X polymorphisms. In a model including resistin levels and Trp64Arg with C1431T polymorphisms, the highest TG level was detected in the presence of Trp64Trp-T1431X polymorphisms and abdominal fat distribution. The analysis of Arg64/X and T3141/X was not considered because of a small group size.

**Table 5:** Serum resistin and metabolic parameters in analyzed polymorphisms Pro12, ALA, TRP1431, and C4131T and the distribution of body fat

Analyzed Polymorphism	Type of obesity	*n*	Resistin	Insulin (mU dL^−1^)	TG (mg dL^−1^)	Significant association between resistin, metabolic parameters, polymorphisms, and type of obesity
			*X* ± SD	*X* ± SD	*X* ± SD	*p*-value
Pro12Pro C1431C	Gynoidal	G1 (*n* = 41)	2.56 ± 2.61	8.19 ± 4.48	108.7 ± 48.4	0.045
	Visceral	G2 (*n* = 34)	2.91 ± 3.62	11.86 ± 6.06	145.6 ± 61.5	
Pro12Pro T1431	Gynoidal	G3 (*n* = 17)	3.06 ± 2.8	7.03 ± 3.5	87.8 ± 35	
	Visceral	G4 (*n* = 7)	2.29 ± 1.5	12.05 ± 4.62	162 ± 42	
Ala12/X C1431C	Gynoidal	G5 (*n* = 13)	2.52 ± 2.34	8.22 ± 2.95	105.3 ± 23.4	
	Visceral	G6 (*n* = 18)	3.33 ± 3.49	9.75 ± 4.43	127 ± 38.2	
Ala12/X T1431	Gynoidal	G7 (*n* = 8)	5.04 ± 5.8	8.61 ± 4.28	100.5 ± 35.9	
	Visceral	G8 (*n* = 9)	4.43 ± 5.94	14.68 ± 12.72	178.1 ± 95.6	
		Comparisons between groups	ns	G1 vs. G8* and G3 vs. G8*	G1 vs. G2* and G1 vs. G8** and G2 vs. G3** and G3 vs. G4* and G3 vs. G8*** and G6 vs. G8* and G7 vs. G8 *	
		Power of the test	<50 %	50 %	50 %	
Trp64Trp C1431C	Gynoidal	G1 (*n* = 43)	2.28 ± 1.45	8.15 ± 4.34	106 ± 34.3	0.027
	Visceral	G2 (*n* = 45)	2.56 ± 2.49	10.62 ± 4.66	136.4 ± 55.5	
Trp64Trp T1431	Gynoidal	G3 (*n* = 21)	3.61 ± 4.16	7.39 ± 3.42	88.1 ± 33	
	Visceral	G4 (*n* = 13)	3.74 ± 5.15	14.11 ± 10.65	166.5 ± 78.4	
Arg64/X C1431C	Gynoidal	G5 (*n* = 11)	3.76 ± 4.99	8.35 ± 3.39	115.2 ± 70.6	
	Visceral	G6 (*n* = 7)	6.27 ± 6.99	14.41 ± 9.65	157 ± 50.6	
		Comparisons between groups	ns	G1 vs. G4** and G3 vs. G4** and G3 vs. G6*	G1 vs. G4** and G2 vs. G3** and G3 vs. G4** and G3 vs. G6*	
		Power of the test	<50 %	50 %	80 %	

* *p* < 0.05; ** p < 0.01; *** p < 0.001

## Discussion

Analyzed with the χ^2^ test, the distribution of selected genotypes and allele frequencies showed a higher prevalence of the Ala12 allele in viscerally obese women (Table 1). Data by Robitaille et al. reported that the carriers of the Ala allele had a greater waist circumference and fat mass (including subcutaneous and visceral fat) than Pro homozygotes among French Canadians (Robitaille et al. 2003). Additionally, a meta-analysis by Masud and Yebased on 30 independent studies (a total number of 19,136 subjects) showed that BMI was significantly higher in Ala allele carriers compared with Pro allele homozygotes (*p* = 0.019) (Masud et al. 2003). Similarly, in our study we found that women with abdominal fat deposition had higher fat mass and were more often Ala12 allele carriers than women with a gynoid distribution of fat (Table 1). Moreover, viscerally obese women were characterized by worse metabolic profiles—higher levels of glucose, insulin, and lipid profiles (Table 2). Because of the strong association between visceral adipose tissue and insulin resistance, groups of women with abdominal obesity are at higher risk of diabetes mellitus and cardiovascular diseases (Ahima and Flier 2000; Grygiel-Górniak et al. 2015).

The levels of adipocytokines were similar in women with visceral and gynoid fat distribution, which can be explained by the increased body mass and BMI in both groups (Table 2). Women with visceral fat deposition were characterized by higher uric acid level. In many studies, hyperuricemia is strongly associated with obesity and metabolic syndrome and can predict visceral obesity and insulin resistance (Johnson et al. 2009; Hikita et al. 2007). Moreover, xanthine oxidoreductase—the enzyme that produces uric acid from xanthine has a role in adipocyte differentiation and is a crucial upstream regulator of PPAR-γ activity (Cheung et al. 2007). In this study, there was a significant increase in the uric acid level (data not shown in tables) in the group with Ala12/X polymorphism when compared to Pro12Pro polymorphism, but we did not find any association of uric acid level with other polymorphisms (Trp64Arg and C1431T). Conversely to our study, in Chinese nonagenarians and centenarians, serum uric acid levels were not associated with polymorphisms of PPAR gamma (Zhou et al. 2012), while the study of Young et al. revealed that Chinese subjects with C1431-allele carriers had higher serum uric acid level (Yang et al. 2009). The differences between our and Chinese studies can be explained by the different ethnicity, dietary habits, and age of the analyzed subjects.

Lower concentrations of visfatin (difference close to statistical significance) and higher levels of resistin (*p* = 0.022) were observed in women with the Arg64 allele (Table 3). The direct correlation between these cytokines and the Arg64/X variant is not known, but low visfatin and high resistin concentrations are characteristic for obesity state and correlate with the risk of diabetes mellitus (El-Mesallamy et al. 2011; Savage et al. 2001; Norata et al. 2007). Moreover, the Trp64Arg polymorphism of the *ADRB3* gene is associated with the increased body weight and insulin resistance (Oeveren et al. 2001; Oizumi et al. 2001; Burguete-Garcia et al. 2014). In most studies, the Arg64/X variant coexisted with increased body mass (overweight or obesity), decreased insulin sensitivity and glucose control, and raised risk of metabolic syndrome and early onset of diabetes mellitus type 2 (Oeveren et al. 2001; Oizumi et al. 2001; Tamaki et al. 2006). Thus, we were able to assume that the presence of the Arg64/X allele could predispose the postmenopausal women in our study to higher risk of metabolic disorders including metabolic syndrome and diabetes mellitus.

Another cytokine modulated by *PPARgamma*-*2* gene is adiponectin (Maeda et al. 2001), which is lower in obese subjects (Arita et al. 1999). Similarly, in our study, women with higher BMI were characterized by lower plasma levels of adiponectin (*p* = 0.029). Besides this, women with lower BMI had lower visfatin concentrations in serum (*p* = 0.019, Table 3), which is in accordance with other data showing diminished levels of this cytokine after body mass reduction (Haider et al. 2006; García-Fuentes et al. 2007; Botella-Carretero et al. 2008). In contrast, other research has reported that serum visfatin levels were significantly associated with obesity even after adjusting for age, sex, and diabetes (Fukuhara et al. 2005; Sandeep et al. 2007). In our model based on visfatin concentrations (Table 4) subjects with a visceral distribution of fat had a greater body mass and glucose level and this effect was potentiated by the T1431 allele. This fact is in accordance with other data, which showed a correlation between the T1431 allele and higher body mass and BMI (Hsueh et al. 2001; Doney et al.^2002^).

Though resistin has been proposed to be a key between insulin resistin and obesity in animal models (Steppan et al. 2001), the role of resistin in metabolic disorders in humans is not obvious. Some studies have shown its role in diabetes mellitus (Nagaev and Smith 2001) and obesity (Way et al. 2001), but there is no report showing its role in postmenopausal obesity in the context of genetic background (the presence of *PPAR gamma*-*2 and ARDB gene polymorphisms*). In a resistin model (Table 4), higher levels of insulin, glucose, and TG were observed in viscerally obese women with the T1431/X polymorphism. The presence of the T1431 allele may increase the risk of metabolic disorders (Doney et al. 2002; Hsueh et al. 2001) and this is associated with significantly increased levels of TG (Gu et al. 2014).

In this study we analyzed the influence of more than one polymorphism on cytokine levels and metabolic disorders. Most studies have described the influence of single polymorphism on adipocytokine levels, for example data by Kim et al. revealed that weight, BMI, and WHR were significantly higher if the Ala allele was present (Kim et al. 2004). In our study, we found that the co-existence of Ala12/X with T1431/X polymorphism was associated with the highest resistin and TG levels (Table 5). Thus, the presence of Ala12/X polymorphism seems to predict “unbeneficial” metabolic changes and is potentiated by the T1341 allele.

The analysis of resistin levels and two other polymorphisms (Trp64Arg and C1431T) showed the differences in TG concentration between women with visceral and gynoid distributions of fat. The highest TG concentration was observed in visceral women with the Trp64Trp allele and the T1431 allele. The T1431 allele is associated with high TG levels (Gu et al. 2014) and increases the risk of metabolic disorders (Doney et al. 2002; Hsueh et al. 2001). Besides this, abdominal fat is one of the main predictors of lipid disorders (Cullen 2000) and cardiac events (Rosenquist et al. 2015). Thus, analyzed viscerally obese women (especially with T1431 allele) could be a monitor of diabetic and cardiac risk.

## Conclusion

The metabolic disorders of analyzed postmenopausal women were determined by their visceral fat distribution. Higher frequencies for the Ala12 allele were observed in visceral subjects, while the Arg64 allele was associated with higher resistin levels. The unbeneficial metabolic changes in carriers with Ala12 allele were potentiated by the T1431 allele. Women with the T1341 allele and a visceral distribution of fat were characterized by higher TG concentrations, independent of co-existing polymorphisms. Considering metabolic risk we can suspect that the analyzed overweight and obese postmenopausal women with T1431 variant could be a monitor of metabolic syndrome.
